# Spondyloenchondrodysplasia With Immune Dysregulation, but Without Skeletal Dysplasia, in a Six-Year-Old Boy: A Case Report

**DOI:** 10.7759/cureus.60314

**Published:** 2024-05-14

**Authors:** Faten Al-Kateb, Duaa Dyab, Basher Almadani, Nora Al-Enezi

**Affiliations:** 1 Pediatrics, King Fahad Medical City, Riyadh, SAU; 2 Pediatrics, Alfaisal University College of Medicine, Riyadh, SAU

**Keywords:** hemolytic anemia, thrombocytopenia, neurological involvement, short stature, immune dysregulation, immune-osseous dysplasia, case report

## Abstract

Spondyloenchondrodysplasia with immune dysregulation (SPENCDI) is a rare autosomal recessive genetic disorder caused by a homozygous mutation of the ACP5 gene. Spondyloenchondrodysplasia is a type of immune-osseous dysplasia manifesting with skeletal dysplasia, immunologic dysfunction, and neurological manifestations. We report the case of a six-year-old boy with SPENCDI who presented with post-viral illness Coombs-positive hemolytic anemia, thrombocytopenia, and fever, based on which he was diagnosed with Evans syndrome. He was previously diagnosed with spastic diplegia, short stature, and celiac disease. The diagnosis was confirmed with genetic testing which displayed a homozygous frameshift mutation of the ACP5 gene c.549del p.(Gln184Serfs*28). This case report discusses the clinical presentation of SPENCDI and highlights the importance of considering this rare genetic disorder in patients presenting with short stature, immunologic dysregulation, and neurological involvement.

## Introduction

Spondyloenchondrodysplasia with immune dysregulation (SPENCDI) is defined as an autosomal recessive genetic disorder caused by ACP5 gene mutation, which mainly affects the skeletal and the immunologic systems' function, along with neurological impairment [1,2]. Spondyloenchondrodysplasia is classified as a type of immune-osseous dysplasia. Skeletal dysplasia includes platyspondyly, metaphyseal dysplasia, and lesions on the spine and long bones, in addition to noncancerous intraosseous cartilage growths (enchondromas); these contribute to the manifestations of short statute, rhizomelia, and increased lumbar lordosis with facial anomalies [1-5]. The dysregulated immune response can manifest with immunodeficiency, which presents with recurrent infections and fever, or autoimmunity that leads to thrombocytopenia, hemolytic anemia, or chronic inflammatory conditions such as systemic lupus erythematosus (SLE) or rheumatoid arthritis (RA) [6,7]. The neurologic manifestations can include spasticity, ataxia, or intellectual disability, and they may also present with intracranial calcifications [1,2]. A case is presented of a six-year-old boy with SPENCDI due to a homozygous frameshift mutation of the ACP5 gene c.549del p.(Gln184Serfs*28) likely pathogenic (Class 2).

## Case presentation

A six-year-old boy who was a known case of spastic diplegic cerebral palsy and celiac disease presented to the emergency department (ED) of our hospital with complaints of gross hematuria for two days following a viral infection. Investigations showed hemolytic anemia and thrombocytopenia; therefore, the patient was admitted as a suspected case of Evans syndrome. He was previously healthy until he suddenly developed a fever reaching 39℃ responding to antipyretics and then developed Cola-colored dark urine, but no dysuria, or change in the smell or the amount of urine, associated with petechial rash over his lower limbs. There was a history of lower leg pain without arthritis. There were also fatigue, pallor, and decreased activity. 

At the age of one year, he had a similar presentation where he presented with a fever that lasted for three weeks and was suspected to be due to an upper respiratory tract infection (URTI); he received antibiotics, packed red blood cells, and platelet transfusion. However, there was no admission or need for oxygen supplement. He recovered from the illness but was noted to not be able to stand as before. After a few months, he started standing but was noted to have tiptoeing and needed assistance to walk a few steps. The patient was surgically free and up to date with his vaccinations. He shares his family's diet and has normal development with only gross motor regression started at the age of 18 months following a viral URTI, when he had lost his ability to stand and walk like before.

Past medical history included spastic diplegia and celiac disease diagnosed at the age of three years and confirmed by biopsy, advised for gluten exclusion diet but was not fully compliant, had short stature due to growth hormone deficiency, and commenced on growth hormone. Family history was remarkable with one sibling who has been recently diagnosed with celiac disease.

At presentation, the patient was active and alert, with a normal appearance. He was also vitally stable except for tachycardia reaching 129 bpm. There was a purpuric rash over his lower limbs and buttocks. He could stand and walk for a few steps with support, tiptoeing wearing his ankle-foot orthoses, but has normal cognition and speech.

Laboratory studies showed markedly low hemoglobin and platelet levels with elevated erythrocyte sedimentation rate (ESR), normal white blood cells, and normal liver and renal function studies with significantly elevated lactate hydrogenase. Additionally, antistreptolysin O (ASO) titers came out negative with normal C3 and C4 serum levels (Table 1).

**Table 1:** Laboratory tests of the patient ESR: erythrocyte sedimentation rate; INR: international normalized ratio; APTT: activated partial thromboplastin time; WBC: white blood cell; Hg: hemoglobin; K: potassium; Na: sodium

Laboratory tests
Albumin: 41
ESR: 36
INR: 1.05
Reticulocyte count: 4.3
Lactate dehydrogenase: 2400
APTT: 30
WBC: 12.3
Hg: 7.1
Platelet: 48
K: 3.7
Urea: 5.2
Na: 137

Urine dipstick showed +++ hemoglobin (Table 2).

**Table 2:** Urine dipstick of the patient WBC: white blood cell; RBC: red blood cell

Urine dipstick
WBC: 19
RBC: 136

The nasopharyngeal airway (NPA) was positive for adenovirus and coronavirus. All other biochemical and microbiological studies were unremarkable. The direct Coombs test was positive (Table 3).

**Table 3:** NPA, other biochemical and microbiological studies, and the direct Coombs test of the patient NPA: nasopharyngeal airway

Result	Test
Positive	Coronavirus OC43
Positive	Adenovirus
Positive	Direct Coombs test
Negative	C3 and C4

At the time of admission, the patient received one pint of packed red blood cells. There was an improvement in hemoglobin levels. The initial hematologist plan was to start intravenous immunoglobulin (IVIG) (human) 10% (Privigen) infusion of 1 g/kg for 2-3 days with an assessment for adverse reactions; after the IVIG course is done, the patient is to be started on methylprednisolone sodium succinate (Solu-Medrol) 2 mg/kg for three days and then shifted to oral prednisone and followed up with red blood cell morphology. Following the first dose of IVIG, the patient had tachypnea and tachycardia which resolved. Due to fever spikes, he received antibiotics after blood and urine tests had been repeated. While the blood tests improved, their values haven't returned to normal, and urine culture was negative, so IVIG was extended for five days, methylprednisolone was continued for another three days, and rituximab was planned but not added. He was also maintained on good hydration to prevent pigment-induced acute kidney injury.

**Figure 1:**
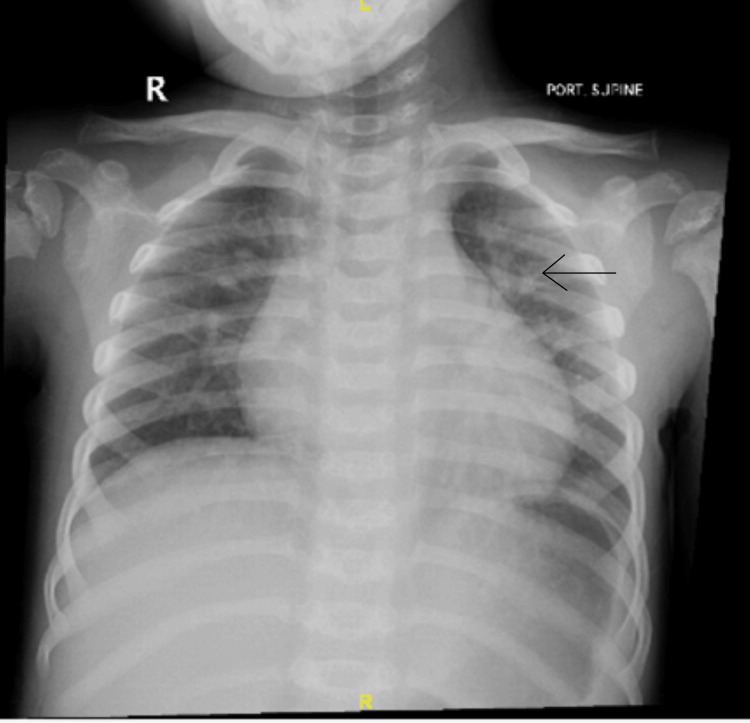
Chest X-ray Left retrocardiac airspace opacity which could represent consolidation/atelectasis, no pneumothorax or pleural effusion with normal cardiac silhouette.

**Figure 2:**
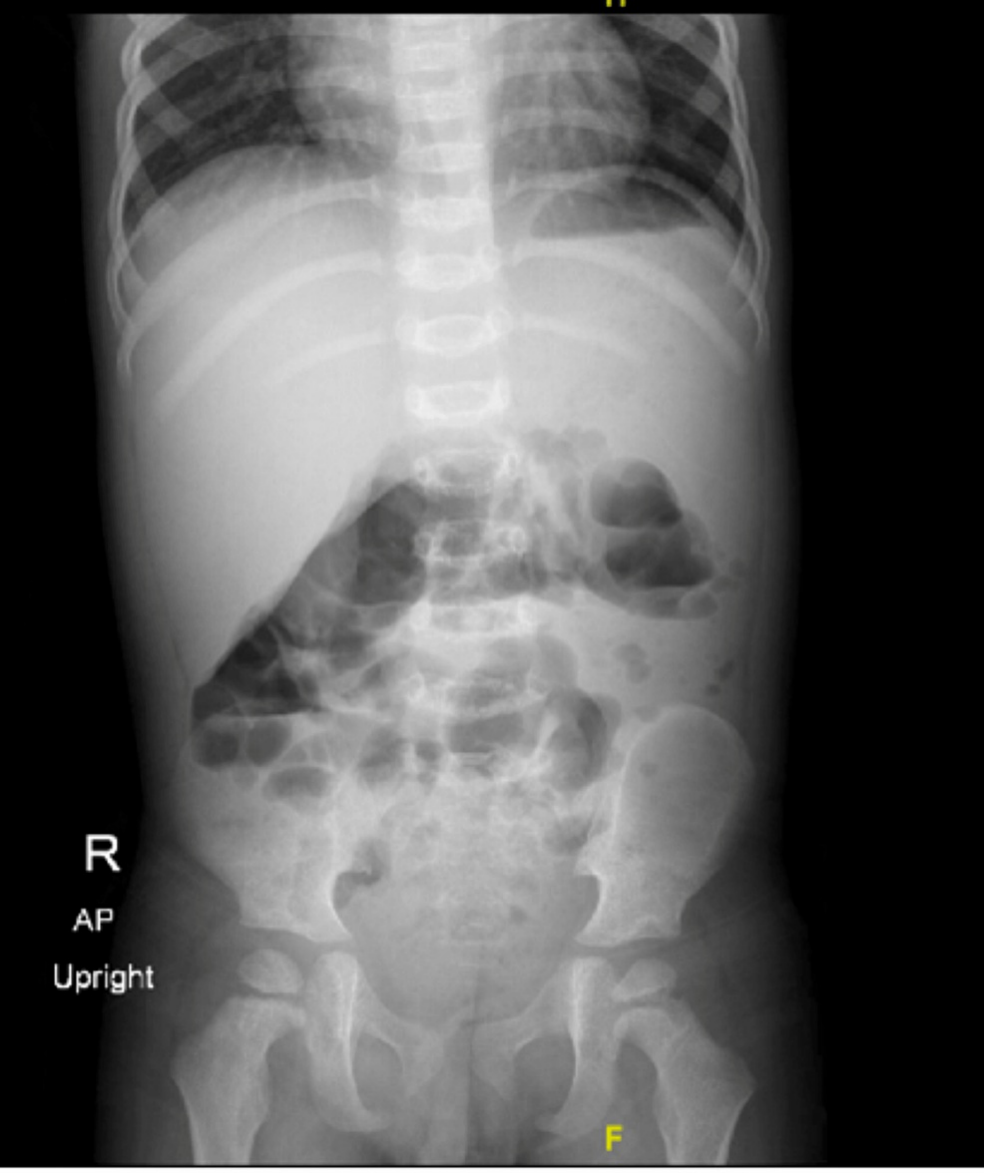
Abdominal X-ray Bowel gas is present in a nonobstructive pattern with no pneumoperitoneum shown.

A chest X-ray (Figure 1) was done at the time of admission and was unremarkable. An abdominal ultrasound was also performed, and it was normal other than echogenic kidneys with mild bilateral distension of the collecting system. He later developed mild abdominal pain, and an abdominal X-ray showed no abnormalities (Figure 2).

On the same day, the patient developed a headache with photophobia and hypertension, and he maintained a normal level of consciousness and reactive pupils despite the oxygen (O2) desaturation and poor limb perfusion. Hydralazine and intravenous (IV) paracetamol normalized his blood pressure but only slightly improved the headache. Aseptic meningitis was suspected especially after the patient had received five doses of IVIG; therefore, a full and meticulous neurological evaluation was done which showed no new findings over his baseline. To rule out intracranial bleeding, a brain computed tomography (CT) scan was requested, and a lumbar puncture was planned if CT results were normal to exclude infectious meningitis; however, lumbar puncture was not done.

**Figure 3:**
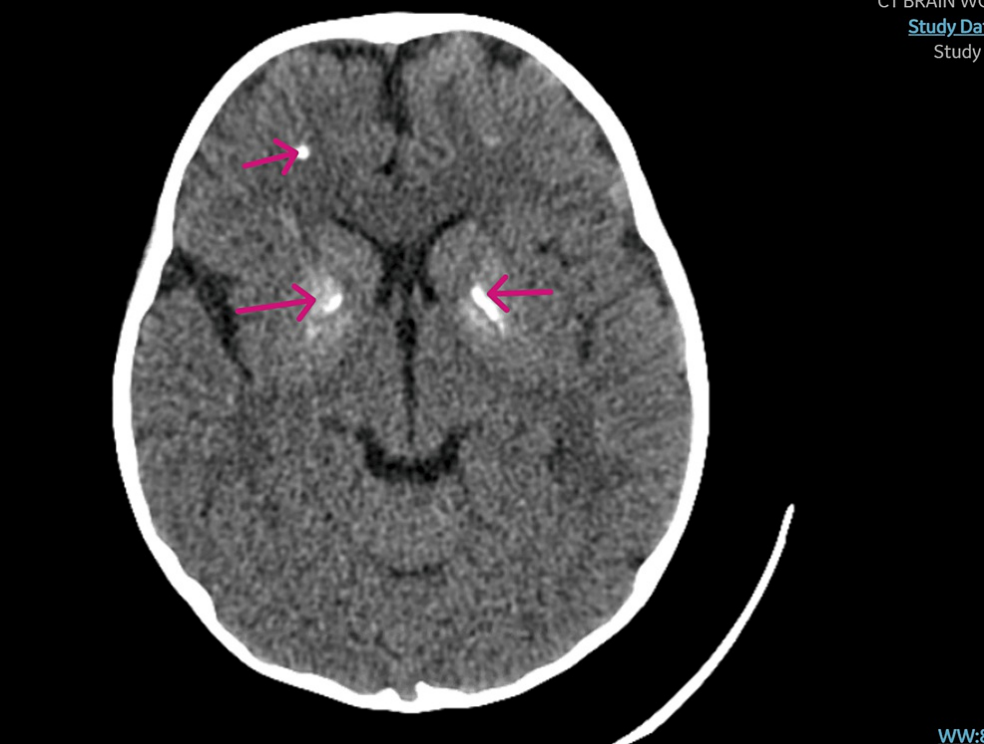
CT scan of the brain, horizontal view No acute well-established territorial infarction or acute intracranial hemorrhage, no mass effect or brain herniation, but multiple calcifications are noted within the basal ganglia, subcortical mainly at the frontal lobe with appropriate size and configuration of the ventricular system and CSF spaces. Posterior structures are grossly unremarkable with the imaging displaying the impression of no acute brain insult. CT: computed tomography; CSF: cerebrospinal fluid

**Figure 4:**
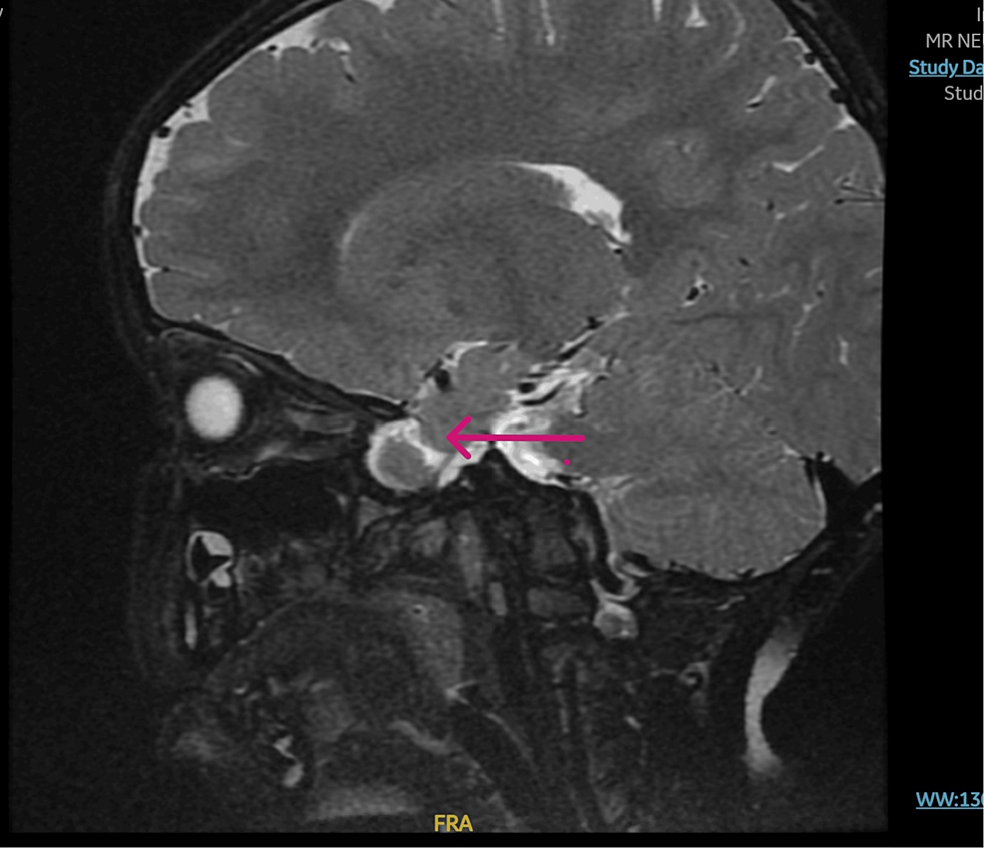
MRI of the brain, sagittal view Subtle bilateral predominately frontal subcortical foci of abnormal susceptibility, likely representing calcifications and suggestive of a remote brain insult with no associated volume loss or other lesions. The brain otherwise demonstrates a normal appearance with preserved volume and signal intensity of the basal ganglia, thalami, brainstem, and cerebellum, and the volume of the pituitary gland is towards the lower limit of normal size with no definite focal lesion, and the pituitary stalk and hypothalamus appear normal. MRI: magnetic resonance imaging

The patient didn't have any signs of posterior reversible encephalopathy syndrome (PRES) on the CT scan (Figure 3), and no brain edema, bleeding, or hemorrhage with multiple calcifications was noted within the basal ganglia. Within an hour, he developed a sudden attack of vision loss associated with respiratory distress and oxygen desaturation that required a non-rebreathing mask. A chest X-ray was done and was unremarkable. Peripheral blood smear did not show any signs of thrombotic thrombocytopenic purpura. A dose of fresh frozen plasma was given to the patient, and urgent magnetic resonance imaging (MRI) (Figure 4) and magnetic resonance angiography (MRA) were ordered, and the patient was shifted to the pediatric intensive care unit (PICU). His clinical situation deteriorated, and he went into cardiac arrest. 

There was no apparent explanation for this prompt deterioration, but it was assumed to be most likely due to cerebral venous thrombosis or pulmonary embolism that led to a rapid and sudden worsening of the clinical condition, which didn't revive with CPR, and might have resulted from a hypercoagulable state.

Panel genetic testing (sequencing and next-generation sequencing (NGS)-based copy number variation (CNV) analysis) was requested during the admission, where a homozygous likely pathologic variant was identified in the APC5 gene (Table 1). This finding is consistent with the genetic diagnosis of autosomal recessive SPENCDI.

ACP5, c.549del p.(Gln184Serfs*28)

The ACP5 variant c.549del p.(Gln184Serfs*28) creates a shift in the reading frame starting at codon 184. The new reading frame ends in a stop codon 27 position downstream. It is classified as likely pathogenic (Class 2) according to the recommendations of CENTOGENE and the American College of Medical Genetics and Genomics (ACMG).

**Table 4:** Sequence variants Variant annotation based on OTPA (VEP v94). * Align-GVD: C0: least likely to interfere with function, C65: most likely to interfere with function; splicing predictions: Ada and RF scores. ** gnomAD, ESP, 1000G, and CentoMD® (latest database available). *** based on ACMG recommendations gnomAD: Genome Aggregation Database; ESP: Exome Sequencing Project; 1000G: 1000Genome project; ACMG: American College of Medical Genetics and Genomics

Gene	Variant coordinates	Amino acid change	SNP identifier	Zygosity	In silico parameters*	Allele frequencies**	Type and classification***
ACP5	NM_001111034.2: c.549del	p.(Gln184Serfs*28)	N/A	Homozygous	PolyPhen: -	gnomAD: -	Frameshift likely pathogenic (Class 2)
					Align-GVDG: N/A	ESP: -	
						1000G: -	
					SIFT: N/A		
					MutationTaster: N/A		
						CentoMD: -	
					Conservation_nt: N/A		
					Conservation_aa: N/A		

## Discussion

SPENCDI, a type of immune-osseous dysplasia, typically manifests with skeletal abnormalities including short stature, thoracolumbar kyphoscoliosis, and rhizomelia with facial dysmorphism. It is an autosomal recessive condition caused by genetic mutations in the ACP5 gene that is responsible for providing instructions for making the enzyme tartrate-resistant acid phosphatase type 5 (TRAP) produced in immune cells and osteoclasts [8]. 

This is a case of a six-year-old boy who presented with Evans syndrome, short stature, and spastic diplegia, the constellation of symptoms that has raised the suspicion of this genetic disorder. Genetic testing revealed the presence of a homozygous frameshift gene mutation of the ACP5 gene c.549del p.(Gln184Serfs*28) likely a pathologic variant (Class 2) that confirmed the diagnosis of SPENCDI [6,9,10].

This case report presents a variant of the variable phenotypical presentations of SPENCDI [11], and it underlies the imperative consideration of this rare genetic diagnosis in patients presenting with brain calcification, significant autoimmunity, and neurological involvement, even without apparent skeletal dysplasia. It also presents a case of the very few previously described cases of the same condition, with proven growth hormone deficiency, as a contributing factor for short stature observed in the syndrome. A case report on a three-year-old patient who presented with spondyloenchondrodysplasia and short stature revealed that growth hormone therapy was beneficial in accelerating growth velocity [12]. 

In comparison to the previous literature, many of which displayed a similar presentation as our patient [2,4,10,11] due to the ACP gene mutation, this case revealed a novel variant which is c.549del p.(Gln184Serfs*28) likely a pathologic variant (Class 2). A comprehensive survey [6] named "Spondyloenchondrodysplasia due to mutations in ACP5" showed that biallelic ACP5 mutations are primarily linked to skeletal, neurological, and immune manifestations, which can vary greatly in their presentation and severity suggesting that this variation may extend to the absence of autoimmune disease, implying that SPENCD and SPENCDI exist on a spectrum of the same condition.

To enhance understanding of the genetic and environmental factors contributing to the phenotypic variability of spondyloenchondrodysplasia, more research is required. This research will help to improve the diagnosis and management of this rare disorder.

Future directions

Further research directions in spondyloenchondrodysplasia include deepening our understanding of the environmental and genetic factors leading to the disease's phenotypical variability, including detecting further factors that contribute to the variable presentation of the condition and recognizing their major role in the disease expression. This can be further achieved by advanced methods such as epigenomics, genome-wide association studies, and environmental exposome studies. 

Also, further research should focus on creating more innovative approaches for diagnosis and follow-up by implementing advanced techniques and procedures facilitating a better understanding of the disease course and a better prognosis. Therefore, research needs to prioritize creating new management strategies for the disease, particularly on addressing immune dysregulation and its related complications for better outcomes.

## Conclusions

Our case represents a six-year-old boy with SPENCDI without skeletal dysplasia. This case report illustrates the importance of considering spondyloenchondrodysplasia in the differential diagnosis of patients presenting with short stature and immune dysfunction. It also remarks on the unique and complex presentation of this rare condition.

The clinical course was characterized by autoimmune pancytopenia, recurring infections, and chronic inflammation. Our patient did not have skeletal dysplasia, despite having ACP gene mutations, suggesting that there is a wide range of phenotypic severity in spondyloenchondrodysplasia. This case shows the significance of early diagnosis and treatment of spondyloenchondrodysplasia and its implication in improving the prognosis.
